# Congenital Heart Disease in Asymptomatic Neonates with Extra-Cardiac Malformations and Genetic Disorders

**DOI:** 10.4274/balkanmedj.galenos.2019.2019.9.104

**Published:** 2019-10-28

**Authors:** Ageliki A. Karatza, Olga Panagiotopoulou, Despoina Gkentzi, Gabriel Dimitriou

**Affiliations:** 1 Department of Pediatrics, University of Patras Medical School, Patras, Greece

To the Editor,

The incidence of congenital heart disease is fairly high in neonates with heart murmurs, ranging from 22% to 86% across studies (1). Therefore, low threshold of referral is recommended in this patient population. A cardiac murmur is identified in approximately 2% of routine neonatal examinations (2). The absence of a murmur does not preclude congenital heart disease. Indeed, more than half of the neonates with congenital heart disease diagnosed in infancy are missed on routine neonatal examination and more than one third are missed at the 6th-week examination (3). Congenital heart disease is associated with extra-cardiac malformations, chromosome abnormalities, and clinical syndromes (4). According to the scientific statement of the American Heart Association, the presence of a chromosome abnormality or extra-cardiac malformation is an indication for fetal echocardiography (4). The rationale behind this recommendation is that earlier diagnosis of congenital heart disease is associated with better outcomes. Of note, infant mortality due to congenital heart disease has significantly reduced in the past few decades, whereas adult congenital heart disease prevalence has increased and is expected to rise (5).

The aim of our study was to determine the prevalence of extra-cardiac malformations, chromosome abnormalities, and clinical syndromes in neonates with congenital heart disease as well as the detection rate of congenital heart disease in this population at the preclinical stage. Over a period of 5.5 years, 866 neonates (1-28 days old) were referred for echocardiography, of which 305 (35.2%) had congenital heart disease. Of these 305 patients, 259 (85%) had isolated congenital heart disease. Hemodynamically significant congenital heart disease was diagnosed in 14 out of 259 (5.4%) patients, an incidence similar to that previously reported (1). Of the remaining 46 neonates with congenital heart disease, 19 (6.2%) had chromosomal abnormalities, 5 (1.6%) had clinical syndromes, and 22 (7.2%) had extra-cardiac anomalies. In 21 out of 46 of these infants (45.6%), the indication for echocardiography was based on identification of an extra-cardiac lesion or suspected clinical syndrome. Of the total cohort, 138 neonates out of 866 had extra-cardiac abnormalities, chromosomal abnormalities, or identifiable syndromes. Presence of any of these factors resulted in a positive predictive value of 33.3% for the co-existence of congenital heart disease. In infants with chromosomal aberrations, trisomy 21 was most common (13/24, 51.2%) and the most prevalent anomaly was an atrioventricular septal defect (7/24, 21.2%), whereas in infants with extra-cardiac anomalies, the most common congenital heart disease was a ventricular septal defect (11/22, 50%). Anomalies of the gastrointestinal tract were the most prevalent in the latter group (7/22, 31.8%).

Based on the results of our study and previously published findings, guidelines should consider incorporating echocardiography prior to discharge from the normal newborn nursery in asymptomatic neonates with extra-cardiac anomalies and genetic diseases even in the absence of a murmur to ensure earlier diagnosis of congenital heart disease.
